# Systemic inflammation is associated with incident stroke and heart disease in East Asians

**DOI:** 10.1038/s41598-020-62391-3

**Published:** 2020-03-27

**Authors:** Mohd A. Karim, Christiana Kartsonaki, Derrick A. Bennett, Iona Y. Millwood, Michael R. Hill, Daniel Avery, Zheng Bian, Huaidong Du, Yu Guo, Yijian Qian, Chan Qu, Iain Turnbull, Dan Schmidt-Valle, Chunmei Wang, Canqing Yu, Jun Lv, Junshi Chen, Robert Clarke, Liming Li, Zhengming Chen, Michael V. Holmes, Robin G. Walters, China Kadoorie Biobank Collaborative Group, Rory Collins, Depei Liu, Richard Peto, Ruth Boxall, Yumei Chang, Yiping Chen, Simon Gilbert, Alex Hacker, Andri Iona, Rene Kerosi, Ling Kong, Om Kurmi, Garry Lancaster, Sarah Lewington, Kuang Lin, John McDonnell, Qunhua Nie, Paul Ryder, Sam Sansome, Paul Sherliker, Rajani Sohoni, Becky Stevens, Jenny Wang, Lin Wang, Neil Wright, Ling Yang, Xiaoming Yang, Pang Yao, Xiao Han, Can Hou, Pei Pei, Chao Liu, Zengchang Pang, Ruqin Gao, Shanpeng Li, Shaojie Wang, Yongmei Liu, Ranran Du, Liang Cheng, Xiaocao Tian, Hua Zhang, Yaoming Zhai, Feng Ning, Xiaohui Sun, Feifei Li, Silu Lv, Junzheng Wang, Wei Hou, Mingyuan Zou, Shichun Yan, Xue Zhou, Bo Yu, Yanjie Li, Qinai Xu, Quan Kang, Ziyan Guo, Dan Wang, Ximin Hu, Jinyan Chen, Yan Fu, Xiaohuan Wang, Min Weng, Zhendong Guo, Shukuan Wu, Yilei Li, Huimei Li, Ming Wu, Yonglin Zhou, Jinyi Zhou, Ran Tao, Jie Yang, Jian Su, Fang liu, Jun Zhang, Yihe Hu, Yan Lu, Liangcai Ma, Aiyu Tang, Yujie Hua, Jianrong Jin, Jingchao Liu, Zhenzhu Tang, Naying Chen, Ying Huang, Mingqiang Li, Jinhuai Meng, Rong Pan, Qilian Jiang, Jian Lan, Yun Liu, Liuping Wei, Liyuan Zhou, Ningyu Chen, Ping Wang, Fanwen Meng, Yulu Qin Sisi Wang, Xianping Wu, Ningmei Zhang, Xiaofang Chen, Weiwei Zhou, Guojin Luo, Jianguo Li, Xiaofang Chen, Xunfu Zhong, Jiaqiu Liu, Qiang Sun, Pengfei Ge, Xiaolan Ren, Caixia Dong, Hui Zhang, Enke Mao, Xiaoping Wang, Tao Wang, Xi Zhang, Ding Zhang Zhou, Gang Zhou, Shixian Feng, Ling Chang, Lei Fan, Yulian Gao, Tianyou He, Huarong Sun, Pan He, Chen Hu, Xukui Zhang, Huifang Wu, Min Yu, Ruying Hu, Hao Wang, Weiwei Gong, Meng Wang, Kaixu Xie, Lingli Chen, Dongxia Pan, Qijun Gu, Yuelong Huang, Biyun Chen, Li Yin, Huilin Liu, Zhongxi Fu, Qiaohua Xu, Xin Xu, Hao Zhang, Huajun Long, Libo Zhang

**Affiliations:** 1 Clinical Trial Service Unit & Epidemiological Studies Unit (CTSU), Nuffield Department of Population Health, University of Oxford, Oxford, United Kingdom; 2 Medical Research Council Population Health Research Unit (MRC PHRU) at the University of Oxford, Nuffield Department of Population Health, University of Oxford, Oxford, United Kingdom; 3 Chinese Academy of Medical Sciences, Beijing, China; 4 Tongxiang Centre for Disease Control and Prevention, Tongxiang, Zhejiang Province China; 5 NCDs Prevention and Control Department, Liuyang Centre for Disease Control and Prevention, Liuyang, Hunan Province China; 6 Department of Epidemiology, School of Public Health, Peking University Health Science Center, Beijing, China; 7 China National Center for Food Safety Risk Assessment, Beijing, China; 8 National Institute for Health Research Oxford Biomedical Research Centre, Oxford University Hospital, Oxford, United Kingdom; 9 Centre for Disease Control and Prevention, Qingdao Province Shandong, China; 10 Licang Centre for Disease Control and Prevention, Qingdao Province Shandong, China; 11 Centre for Disease Control and Prevention, Heilongjiang Province, Harbin, China; 12 Nangang Centre for Disease Control and Prevention, Heilongjiang Province, Harbin, China; 13 Centre for Disease Control and Prevention, Hainan Province, Haikou, China; 14 Meilan Centre for Disease Control and Prevention, Hainan Province, Haikou, China; 15 Centre for Disease Control and Prevention, Jiangsu Province, Nanjing, China; 16 Suzhou Centre for Disease Control and Prevention, Jiangsu Province, Suzhou, China; 17 Wuzhong Centre for Disease Control and Prevention, Jiangsu Province, Suzhou, China; 18 Centre for Disease Control and Prevention, Guangxi Province, Nanning, China; 19 Liuzhou Centre for Disease Control and Prevention, Guangxi Province, Liuzhou, China; 20 Centre for Disease Control and Prevention, Sichuan Province, Chengdu, China; 21 Pengzhou Centre for Disease Control and Prevention, Sichuan Province Pengzhou, China; 22 Centre for Disease Control and Prevention, Gansu Province, Lanzhou, China; 23 Maiji Centre for Disease Control and Prevention, Gansu Province Tianshui, China; 24 Centre for Disease Control and Prevention, Henan Province Zhengzhou, China; 25 Huixian Centre for Disease Control and Prevention, Henan Province Huixian, China; 26 Centre for Disease Control and Prevention, Zhejiang Province Hangzhou, China; 27 Tongxiang Centre for Disease Control and Prevention, Zhejiang Province Tongxiang, China; 28 Centre for Disease Control and Prevention, Hunan Province Changsha, China; 29 Liuyang Centre for Disease Control and Prevention, Hunan Province Liuyang, China

**Keywords:** Predictive markers, Myocardial infarction, Stroke

## Abstract

Systemic inflammation, reflected by increased plasma concentrations of C-reactive protein (CRP) and fibrinogen, is associated with increased risk of coronary heart disease, but its relevance for stroke types remains unclear. Moreover, evidence is limited in non-European populations. We investigated associations of CRP and fibrinogen with risks of incident major coronary events (MCE), ischemic stroke (IS) and intracerebral hemorrhage (ICH) in a cohort of Chinese adults. A nested case-control study within the prospective China Kadoorie Biobank included 1,508 incident MCE cases, 5,418 IS cases, 4,476 ICH cases, and 5,285 common controls, aged 30–79 years. High-sensitivity CRP and low-density lipoprotein cholesterol (LDL-C) were measured in baseline plasma samples from all participants, and fibrinogen in a subset (n = 9,380). Logistic regression yielded adjusted odds ratios (ORs) per SD higher usual levels of log-transformed CRP and fibrinogen. The overall mean (SD) baseline LDL-C was 91.6 mg/dL (24.0) and geometric mean (95% CI) CRP and fibrinogen were 0.90 mg/L (0.87–0.93) and 3.01 g/L (2.98–3.03), respectively. There were approximately log-linear positive associations of CRP with each outcome, which persisted after adjustment for LDL-C and other risk factors, with adjusted ORs (95% CI) per SD higher CRP of 1.67 (1.44–1.94) for MCE and 1.22 (1.10–1.36) for both IS and ICH. No associations of fibrinogen with MCE, IS, or ICH were identified. Adding CRP to prediction models based on established risk factors improved model fit for each of MCE, IS, and ICH, with small improvements in C-statistic and correct reclassification of controls to lower risk groups. Among Chinese adults, who have low mean LDL-C, CRP, but not fibrinogen, was independently associated with increased risks of MCE and stroke.

## Introduction

Coronary heart disease (CHD) and stroke are the leading causes of premature death globally^1^. In addition to traditional risk factors, such as smoking, blood pressure and LDL-C^2^, there is accumulating evidence that inflammation plays an important role in the etiology of cardiovascular disease (CVD)^3^.

The local inflammatory response following injury to the arterial endothelium is regarded as a key step in the initiation of atherosclerotic CVD^4^. Injury triggers a cascade that recruits immune cells (monocytes and macrophages), promotes oxidation and aggregation of low-density lipoprotein cholesterol (LDL-C) and contributes to development of atherosclerotic plaques^5,6^. The process may be amplified by elevated levels of circulating pro-inflammatory cytokines^7^ and, hence, persistent low-grade systemic inflammation has emerged as an important modifiable risk factor for atherosclerosis^8,9^.

Circulating C-reactive protein (CRP) and fibrinogen, regarded as markers of systemic inflammation, are associated with increased risk of atherosclerosis^10–16^ and atherosclerotic CVDs including CHD and ischemic stroke (IS)^17–21^. Most of the available evidence has been derived from observational studies of Western populations in whom LDL-C levels are high (mean ~140 mg/dL)^17^. While Mendelian randomization (MR) studies have indicated that CRP and fibrinogen are unlikely to be causal in atherosclerotic CVD^22–26^, such inflammatory markers are nevertheless useful indicators of the activities of pro-inflammatory cytokines that promote their synthesis. For example, the Canakinumab Anti-inflammatory Thrombosis Outcome Study (CANTOS) trial, in which inhibition of the pro-inflammatory cytokine interleukin-1 beta (IL-1β) provided the first evidence from a randomized controlled trial (RCT) for a role of inflammation in CVD^9^, assessed IL-1β activity via measurements of CRP and fibrinogen levels^27^.

Compared to the well-established associations of CRP with risk of CHD, observational evidence concerning the relevance of inflammation to individual stroke types is limited. In particular, there is limited evidence on the associations of systemic inflammation, including CRP and fibrinogen, with risk of intracerebral hemorrhage (ICH). Reliable assessment of the association of systemic inflammation with stroke types is particularly important in China, where stroke rates are much higher than for Western populations, particularly for ICH^28^, in spite of relatively low mean LDL-C levels (~92 mg/dL). To address these questions, we examined the associations of CRP and fibrinogen with risks of incident major coronary events (MCE), IS, and ICH in a nested case-control study, set within the prospective China Kadoorie Biobank (CKB).

## Results

### Baseline characteristics of study participants

Table 1 shows the characteristics of the study participants. Controls (mean age 55.8 years, 49.8% female) were older and had a higher proportion of men than the overall CKB population^29^, reflecting the study design for the nested case-control cohort from which the study population was drawn. Cases of IS (55.4 years) and MCE (56.5 years) were similar in age to controls but ICH cases (59.3 years) were older. Although the proportion of women was similar between controls (49.6%) and ICH cases (49.9%), a lower proportion of women had MCE (41.6%) whilst a higher proportion had an IS event (55.6%). Systolic blood pressure (mmHg) was higher in MCE (143.6) and IS (144.4) cases than in controls (134.4), but was highest in ICH cases (152.9). Compared to controls, larger proportions of MCE and stroke cases reported poor health (MCE: 18%; IS: 15%; ICH: 16%; controls: 8%), hypertension (MCE: 22%; IS: 24%; ICH: 26%; controls: 10%), and diabetes (MCE: 14%; IS: 12%; ICH: 9%; controls: 6%). There were, however, no appreciable differences in adiposity traits (BMI, waist-hip ratio) or levels of self-reported physical activity between cases and controls.

**Table 1:** Baseline traits of study participants.

Trait	Controls (n = 5285)	Major Coronary Events (n = 1508)		Ischemic Stroke (n = 5418)		Intracerebral Hemorrhage (n = 4476)		
**Age and Sociodemographic factors**								
Age, y^*^	55.8 (10.9)		56.5 (9.72)	55.4 (9.36)			59.3 (10.1)	
Female^†^, %	49.6		41.6	55.6			49.9	
Rural^‡^, %	79.2		66.6	54.6			79.5	
Middle School and above, %	38.3		36.8	38.2			36.0	
Income > 10,000 yuan/year, %	62.2		61.5	65.6			62.5	
**Lifestyle factors**								
Male smokers, %	87.1		90.9	89.5			86.9	
Female smokers, %	3.9		7.8	5.4			5.9	
Male regular drinkers, %	32.1		26.5	30.9			33.1	
Female regular drinkers, %	2.1		1.8	2.2			2.1	
Physical activity, MET h/day	20.1 (11.8)		18.7 (11.6)	18.7 (11.5)			19.0 (11.6)	
**Anthropometric, blood pressure and circulatory measures**								
Standing height, m	1.59 (0.06)		1.59 (0.06)	1.59 (0.06)			1.58 (0.06)	
BMI, kg/m^2^	23.4 (3.1)		23.9 (3.4)	24.1 (3.4)			23.8 (3.4)	
Waist-hip ratio	0.89 (0.07)		0.91 (0.07)	0.90 (0.07)			0.90 (0.07)	
SBP, mmHg	134.4 (19.7)		143.6 (25.1)	144.4 (23.9)			152.9 (27.3)	
DBP, mmHg	78.5 (11.0)		83.0 (13.3)	83.9 (12.5)			88.5 (14.2)	
Heart rate, per minute	78.3 (11.7)		80.4 (12.7)	79.2 (12.1)			79.9 (12.6)	
**Markers of subclinical atherosclerosis**								
Plaque, %	39.9		60.5	50.7			44.4	
cIMT, mm	0.71 (0.13)		0.78 (0.23)	0.75 (0.16)			0.72 (0.16)	
**Inflammatory biomarkers**								
CRP, mg/L^§^	0.90 (0.86–0.93)		1.31 (1.24–1.39)	1.12 (1.08–1.16)			1.08 (1.04–1.13)	
Fibrinogen, g/L^§^	3.00 (2.98–3.03)		3.07 (3.04–3.11)	3.05 (3.02–3.08)			3.02 (2.99–3.05)	
**Lipids**								
Triglycerides^§^, mg/dL	145.8 (143.1–148.5)		163.4 (158.5–168.5)		156.6 (153.9–159.45)			145.5 (142.7–148.5)
LDL cholesterol^?^, mg/dL	91.6 (23.9)		95.9 (29.1)		95.9 (25.8)			90.8 (26.4)
**Self-reported health and disease**								
Poor self-rated health, %	8.1		18.4	14.6			15.5	
Hypertension, %	10.4		21.7	23.7			26.4	
Diabetes, %	6.0		14.0	12.4			9.3	

CRP: C-reactive protein; cIMT: carotid intima-media thickness; DBP: Diastolic Blood Pressure; ICH: Intracerebral hemorrhage; IS: Ischemic stroke; LDL: Low-density lipoprotein; MET: metabolic equivalents; SBP: Systolic Blood Pressure.

Values are mean (standard deviation) for continuous traits or proportion (weighted percentage) for binary/categorical traits, standardized to age, sex and study area of case-control cohort unless otherwise stated.

*Standardized to sex and study area only.

^†^Standardized to age and study area only.

^‡^Standardized to age and sex only.

^§^Geometric mean (95% CI). ^?^Note: Low-density lipoprotein (LDL) cholesterol was directly measured.

Markers of inflammation (CRP and fibrinogen), lipids (LDL-C and TG), and subclinical atherosclerosis (proportion of participants with plaque, carotid intima media thickness) were higher in participants with MCE and IS (i.e. atherosclerotic CVD) than in controls. By contrast, differences in these traits were smaller (for CRP and plaque) or absent between ICH cases and controls.

### Cross-sectional associations of CRP and fibrinogen

**Figure 1:**
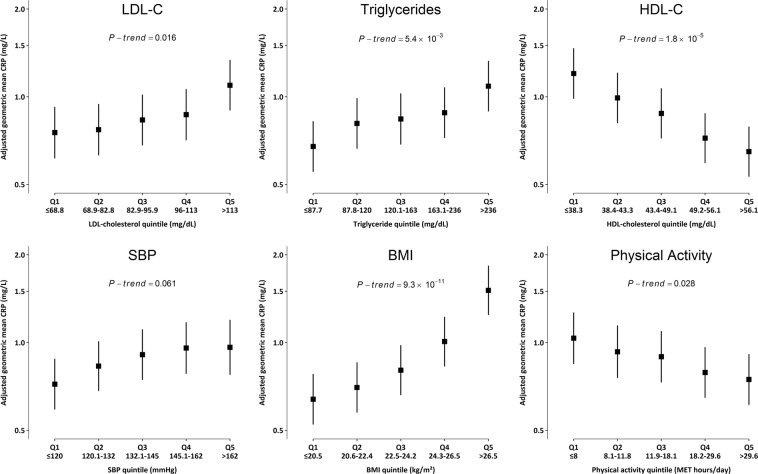
Cross-sectional associations of high-sensitivity C-reactive protein (CRP) with selected cardiovascular disease (CVD) risk factors. Analyses were carried out only in controls (n = 5,285). Cut-offs for biomarker quintiles were based on their distribution in controls only. Geometric mean of CRP was adjusted for age, age^2^, sex and region. *P-trend* was calculated using chi-square test statistic with 1 degree of freedom. Error bars represent 95% CI for each quintile.

Both CRP and fibrinogen showed strong associations with a range of key CVD risk factors and potential confounders. In control individuals there were positive associations of CRP with LDL-C (*P-trend* = 0.016), TG (*P-trend* = 5.3 × 10^−3^), and BMI (*P-trend* = 9.3 × 10^−11^), a less strong positive association with SBP (*P-trend* = 0.06), and inverse dose-response associations with HDL-C (*P-trend* = 1.8 × 10^−5^) and physical activity (*P-trend* = 0.028) (Figs. 1, S2). CRP also showed positive cross-sectional associations with markers of liver (AST, ALT, and GGT) and renal function (creatinine, uric acid, and cystatin-C), and ApoB, and Lp(a), inverse associations with HDL-C, ApoA1 and albumin, and no association with total vitamin D (Fig. S2). A similar pattern of associations was seen for fibrinogen, except that no association was observed for TG. Further cross-sectional associations of CRP and fibrinogen were observed with a wide range of population baseline characteristics, in particular strong positive associations with age, with differences between the top and bottom age categories of 0.50 mg/L (*P* = 1.16 × 10^−126^) for CRP and 0.61 g/L (*P* = 5.42 × 10^−307^) for fibrinogen, representing the impact of a mean difference of 24 years (Table S2).

### Associations of CRP and Fibrinogen with MCE and Stroke types

**Figure 2:**
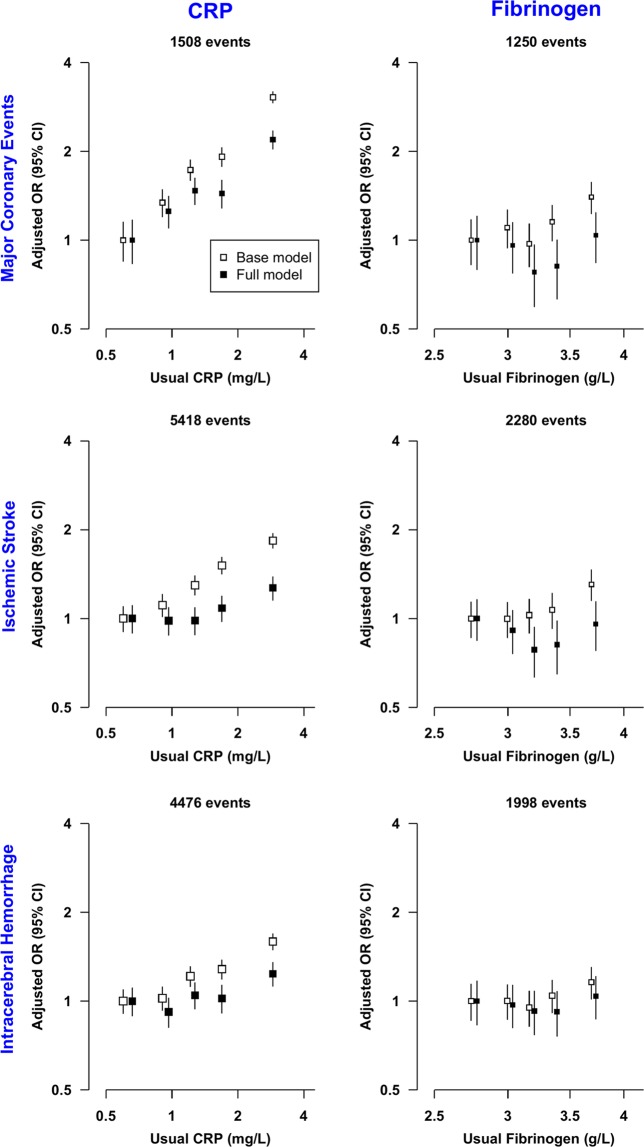
Risk of Major Coronary Events, Ischemic Stroke, and Intracerebral hemorrhage by quintiles of C-reactive protein (CRP) and fibrinogen. Models were adjusted for age, age^2^ and sex (Base model) and additionally for income, occupation, education, SBP, BMI, diabetes, physical activity, standing height, smoking, alcohol, LDL-C and TG (Full model). These models were fitted separately for each of the 10 study areas for each quintile and estimates were meta-analyzed; in each quintile, study areas were excluded when models failed to converge (9 models out of 600 models). Cut-offs for CRP and Fibrinogen quintiles were based on their distribution in the combined case-control cohort (CRP: 0.37 mg/l, 0.70 mg/l, 1.28 mg/l, and 2.59 mg/l; Fibrinogen: 2.47 g/l, 2.81 g/l, 3.14 g/l, and 3.60 g/l). Error bars represent 95% floated confidence intervals (CI). The areas of the boxes are proportional to the inverse of the variance of the log ORs. OR, CRP, and fibrinogen are plotted on log scales.

**Figure 3:**
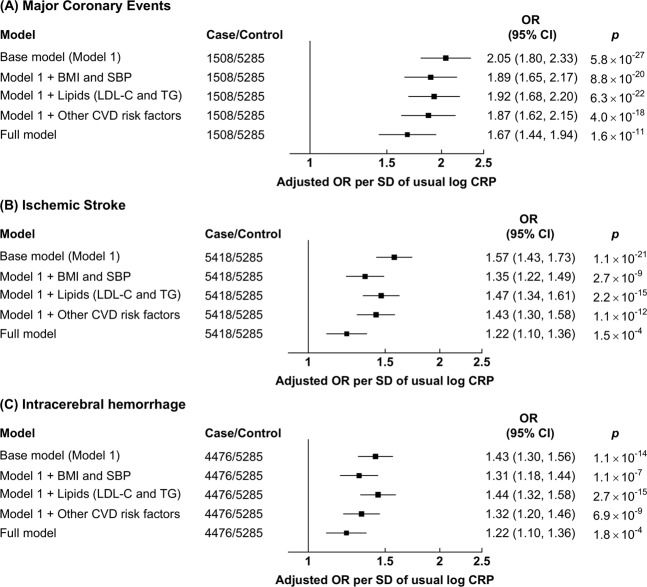
Risk of (**A**) Major Coronary Events, (**B**) Ischemic Stroke, and (**C**) Intracerebral hemorrhage per standard deviation (SD) of usual log-transformed high-sensitivity C-reactive protein (CRP). Models were adjusted for age, age^2^ and sex (base model) and additionally for income, occupation, education, SBP, BMI, diabetes, physical activity, standing height, smoking, alcohol, LDL-C and TG (Full model). The models were fitted separately for each of the 10 study areas and estimates were meta-analyzed. Error bars represent 95% confidence intervals (CI). The areas of the boxes are proportional to the inverse of the variance of the log ORs.

There were log-linear positive associations of log CRP with risks of MCE, IS and ICH (Fig. 2), with adjusted ORs [95% CI] per-SD higher usual log CRP of 2.05 [1.80–2.33] for MCE, 1.57 [1.43–1.73] for IS, and 1.43 [1.30–1.56]) for ICH in the base model (adjusted only for age, age^2^ and sex) (Fig. 3). For each, the association was attenuated after adjustment for SBP, but the association persisted even after further adjustment for BMI, lipids (LDL-C and TG) and other CVD risk factors (Figs. 3, S4(A)). After adjusting for LDL-C the association of CRP with MCE was attenuated to 1.77 [1.54–2.04], and in the fully adjusted model was 1.67 [1.44–1.94]. Similarly, the associations of IS and ICH with CRP after full adjustment were attenuated to 1.22 [1.10–1.36] and (again) 1.22 [1.10–1.36], respectively.

**Figure 4:**
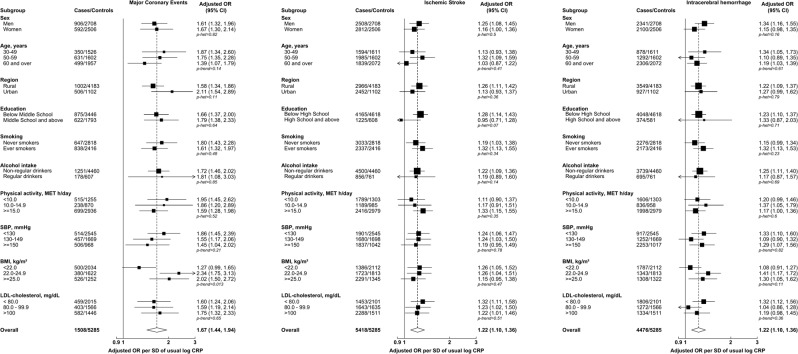
Risk of Major Coronary Events, Ischemic Stroke, and Intracerebral hemorrhage per standard deviation (SD) of usual log-transformed high-sensitivity C-reactive protein (CRP) by pre-specified subgroups. Models were adjusted (where appropriate) for age, age^2^ and sex (base model) and additionally for income, occupation, education, SBP, BMI, diabetes, physical activity, standing height, smoking, alcohol, LDL-C and TG. The models were fitted separately for each of the 10 study areas in each subgroup strata and estimates were meta-analyzed; in each subgroup strata, study areas were excluded when models failed to converge (62 models out of 750 models). Error bars represent 95% confidence intervals (CI). The sizes of the boxes are proportional to the inverse of the variance of the log ORs. The dotted line represents the overall adjusted OR. The open diamond represents the overall adjusted OR and its 95% CI. MET: metabolic equivalent of task; *p-het*: p-value for heterogeneity; *p-trend*: p-value for trend.

We further assessed whether associations of log CRP with MCE, IS and ICH risk differed within pre-specified subgroups of environmental, behavioral, or physiological factors. There was no evidence (after Bonferroni adjustment, *P* < 0.002) for effect modification by any of these factors, including by LDL-C (Fig. 4).

By contrast, although fibrinogen levels were associated with MCE and IS in the base model, the association attenuated to the null after further adjustment (MCE, OR: 1.10 [0.90–1.36]; IS, OR: 1.00 [0.84–1.19], per-SD higher usual log fibrinogen); with ICH, no association was observed, even in the base model (Figs. 2, S3,S4). Associations of fibrinogen with MCE, IS, and ICH risk did not exhibit differences between most of the pre-specified subgroups, including of lipids; however, there was evidence of heterogeneity by levels of physical activity (*P* = 6.7 × 10^−4^) for MCE and suggestive evidence of an interaction with smoking (*P* = 0.0021) for IS (Fig. S5).

### Discriminatory performance

Addition of CRP to a logistic regression model, comprising age, sex, region, smoking, diabetes, BMI, SBP, LDL-C, HDL-C, and TG, yielded small improvements in the C-statistic for MCE (0.7181[95% CI: 0.7028–0.7334] to 0.7251 [95% CI: 0.7101–0.7402]) which were significant by DeLong’s test, but there were smaller differences in the AUCs for IS and ICH (Table S3). Nevertheless, a likelihood ratio test (LRT) - which is a more powerful test of model fit than the rank-based C statistic^30^ - identified improved model fit for MCE (LRT *p* = 1.21 × 10^−13^), IS (LRT *p* = 1.75 × 10^−4^) and ICH (*p* = 9.54 × 10^−7^). Marginal net reclassification improvements (NRI) were observed for MCE (NRI = 0.035 [95% CI: 0.014, 0.056], p = 0.0011]), IS (NRI = 0.011 [95% CI: 0.054, 0.016], p = 8.0 × 10^−5^]), and ICH (NRI = 0.0064 [95% CI: 0.0011, 0.0117], p = 0.019]) (Table S4), showing that in each case the model improvement predominantly reflected improved reclassification of controls to lower risk; a similar improvement in the reclassification of cases was not seen.

## Discussion

In this large nested case-control study of Chinese adults, who have low mean LDL-C (~92 mg/dL), CRP was positively associated with risks of incident MCE, IS, and ICH, independent of established risk factors. The magnitude of the increase in risk was greater for MCE than for stroke, was similar for IS and ICH, and was consistent across different population subgroups. By contrast, after adjustment for other risk factors we observed no increase in risk associated with higher levels of fibrinogen. The findings of this large study extend the well-established association in Europeans of CRP with atherosclerotic disease, and further support a role for systemic inflammation in vascular disease, independent of lipids.

Our findings for CRP are broadly consistent with previous prospective studies conducted in both European and East Asian populations which have consistently demonstrated strong positive associations of CRP with risk of CHD and ischemic stroke^17,19–21^, but with at best marginal improvements in risk prediction^31,32^. In a large meta-analysis of 54 prospective studies in populations of European ancestry, involving 5,373 CHD cases (first-ever MI or fatal CHD) and 1,931 IS cases, each 1 SD higher usual log CRP concentration was associated with 37% and 27% higher risk of CHD and IS, respectively. Our corresponding estimate for IS was very similar (22%), but our MCE estimate (67%) was appreciably greater than in the meta-analysis, with very little overlap of confidence intervals. This perhaps reflects a different spectrum of disease severity and/or aetiology – there were a much higher proportion of fatal MCE cases in the present study (65% vs 29%).

An important rationale for our study was to investigate whether inflammatory markers have different relationships with the pathophysiologically distinct IS and ICH stroke types^33^. Previous studies have highlighted their differing, even opposing, relationships with some CVD risk factors such as LDL-C^34^ or BMI^35^. By contrast, we found associations of CRP with IS and ICH that were concordant in both direction and strength. These results differ from previous studies which reported null associations of CRP with ICH, although these included relatively few ICH cases (383 to 738 in previous studies vs 4,476 in our study), typically leading to effect estimates with wide confidence intervals that overlap our estimate of a 27% increase in risk per SD higher usual log CRP concentration^19–21^. In separate analyses in CKB, other markers of inflammation that are only moderately correlated with CRP, such as glycoprotein acetyls^36^, also exhibited positive associations with both IS and ICH, providing additional support for the role of inflammation in both stroke types^37^.

Unexpectedly, for fibrinogen, associations with both MCE and IS when adjusting only for age and sex were attenuated to the null after further adjustment for a range of potential confounders. A large meta-analysis of 31 prospective studies, involving a total of 154,211 participants (including 7% from Japan), demonstrated positive associations with CHD and overall stroke (mainly IS) which, although partially attenuated, remained strong after adjustment for classical risk factors, including smoking, alcohol, BMI, LDL-C, TG, and SBP, although there remained the possibility of substantial residual confounding^18^. Such residual confounding could potential account for the apparent discrepancy between the two studies. Alternatively, the apparent disparity between the results for CRP and fibrinogen perhaps reflects that they reflect non-identical components of systemic inflammation. This is supported by the strong association in our study between TG and CRP, yet there was no such relationship between TG and fibrinogen. Importantly, the previous meta-analysis that reported an association of fibrinogen with CVD also showed a strong positive association of fibrinogen with TG^18^.

These findings have potential implications for treatment strategies to modify inflammation for prevention of vascular disease^9,38,39^. Importantly, the CANTOS trial of ~10,000 patients with prior MI (>90% on statins, median baseline LDL-C levels in placebo group: 83 mg/dL) demonstrated a beneficial effect of therapeutic inhibition of IL-1β on the primary endpoint of major vascular events. Compared with placebo, allocation to canakinumab was associated with pooled hazard ratios (95%CI) for MI and stroke of 0.84 [0.73–0.97] and 0.93 [0.72–1.20], respectively. While treatment with canakinumab did not lead to a reduction in risk of stroke, this may reflect the lack of power rather than representing a true null effect, since only 264 stroke cases accrued during the 48 month trial duration. Thus, although individuals in the present observational study were drawn from the general population (as opposed to being a study of patients with a previous MI as in the CANTOS interventional trial), were not on statins, and had with a median baseline LDL-C levels (in controls) of 86 mg/dL, our data confirm the potential importance of inflammation pathways for stroke and MCE, independent of LDL-C.

A key strength of our study lies in the large number of well-characterized stroke cases. The majority (90%) had CT or MRI scans at the time of hospital admission^40^; further adjudication by clinician-led review of medical records refuted the diagnosis in only 7% of cases with IS and 8% with ICH. In addition, we measured CRP and fibrinogen using plasma samples of study participants collected at baseline, prior to the occurrence of incident vascular events, thus somewhat limiting reverse causality bias. Further, we were able to correct for measurement error and within-person variation in CRP and fibrinogen levels (i.e. regression dilution bias) by using measurements from repeat plasma samples collected 6–9 years later, so that our results reflect the risk associated with usual levels of these biomarkers.

### Study limitations

The associations of CRP with risk of incident MCE, IS and ICH were attenuated by inclusion of additional risk factors, and it is possible that there remains residual confounding due to inaccurate measures of several such potential confounders (e.g. physical activity), or biases from other sources such as reverse causality. However, the impact of such confounding seems likely to be limited, and the prospective study design likely limits reverse causation. Conversely, the lack of association of fibrinogen with MCE and stroke types in our study may reflect limited statistical power. While, the present study included twice the number of IS cases compared to the largest fibrinogen meta-analysis to date (our study vs meta-analysis, 2,280 vs 1,370 IS cases in fully adjusted models)^18^, we are currently unable to determine the contributions of different stroke sub-types. Associations of CRP and fibrinogen may not be consistent across subtypes of IS (e.g. large artery, small vessel, and cardio-embolic) and ICH (e.g. lobar and non-lobar), something that will be investigated once ongoing characterization of stroke subtypes in CKB is complete.

It should be emphasized that CRP does not play a causal role in stroke, as indicated by large-scale evidence from Mendelian randomization studies^22,24^. Rather, the consistent observed associations of CRP with MCE, IS and ICH likely reflect roles for biomarkers upstream in the inflammatory signaling pathway (e.g. IL-6 or IL-1β), as has been shown previously for CHD and IS^17,41,42^.

## Methods

### Study population

China Kadoorie Biobank is a prospective study of 512,715 adults (aged 30–79 years at enrolment). CKB survey methods and the cohort have been described in detail previously^29,43^. The baseline survey recruited participants between 25 June 2004 and 15 July 2008 from 10 diverse regions (5 urban and 5 rural) of China. Ethical approval for CKB was obtained from the University of Oxford, the Chinese Centre for Disease Control and Prevention (CCDC) and the regional CDC from the 10 study areas and was conducted according to the Declaration of Helsinki. Ethical approval for CKB study was obtained from the University of Oxford (Oxford Tropical Research Ethics Committee), the Chinese Centre for Disease Control and Prevention (CCDC) and the regional CDC from the 10 study areas. All eligible participants provided informed written consent for participation in the CKB study. Release 15 of the CKB database was used for all analyses.

### Data collection

Following informed written consent, study participants (at baseline and resurveys of approximately 5%) were interviewed on socio-demographics (e.g. age, occupation), lifestyle (e.g. smoking, alcohol consumption) and past medical history (e.g. diabetes, hypertension). Anthropometric (e.g. height, weight, hip and waist circumferences) and physiological measurements (e.g. blood pressure) were obtained using calibrated instruments and following a standard protocol. A 10 mL non-fasting blood sample (with last meal time recorded) was collected from all participants at baseline and a subset of study participants during resurveys and stored on ice until processing and cryopreservation.

### Follow up for fatal and non-fatal outcome

Participants were followed up for incident events, using their unique national identification numbers, by electronic linkage to mortality and disease (CHD, stroke, cancer, and diabetes) registries and to a nationwide health insurance system. These events were coded using the *International Classification of Diseases and Related Health Problems, Tenth Revision* (ICD-10). Medical records for hospitalization due to CVD events have been systematically reviewed by clinicians in Oxford and China; approximately 90% of stroke diagnoses were supported by computed tomography (CT) scan or magnetic resonance imagining (MRI)^40^.

### Selection of cases and controls

Study participants included in this study were a subset of a larger nested case-control study of incident stroke and ischemic heart disease (IHD) for which participants were selected in June 2013, comprising those for whom clinical chemistry measurements were available from their baseline plasma samples; individuals with a history of CVD or cancer at baseline were excluded from both cases and controls. One individual with missing data on BMI was also excluded from all analyses (Fig. S1).

Cases were those whose first CVD incident event was a fatal or nonfatal non-traumatic ICH (ICD-10 codes: I61, I69.1), IS (ICD-10 codes: I63, I69.3), or MCE (defined as acute myocardial infarction [ICD-10: I21–I23] or fatal IHD [ICD-10: I20, I24 or I25] or undergoing a coronary revascularization procedure such as stent placement or coronary artery bypass graft) before 1^st^ January 2017. Those who had two or more of MCE, IS and/or ICH events on the same day, or who had had earlier CVD events other than MCE, IS or ICH during follow-up, were excluded. Common controls were those with no CVD event (endpoint definitions are provided in Table S1) before 1^st^ January 2017.

### Measurement of biomarkers

Clinical biochemistry, including markers of inflammation (high-sensitivity CRP [hsCRP], and fibrinogen), lipids (total cholesterol, LDL-C, triglycerides [TG], high-density lipoprotein cholesterol [HDL-C]), lipoproteins (lipoprotein(a) [Lp(a)], apolipoprotein A1 [ApoA1] and apolipoprotein B [ApoB]), markers of liver function (albumin, aspartate transaminase [AST], alanine transaminase [ALT] and gamma-glutamyl transferase [GGT]), markers of renal function (creatinine, uric acid and cystatin-C), and total vitamin D, were assayed at the NDPH Wolfson Laboratory, Oxford. Assays were performed using an AU680 clinical chemistry analyser (Beckman-Coulter, UK) with the following exceptions: plasma fibrinogen and cystatin C were measured using a BN Prospec nephelometer analyser (Siemens, UK); and vitamin D was measured using an Access 2 Immunoanalyser (Beckman-Coulter, UK). All assays used standard manufacturers’ reagents, calibrators, and settings.

Amongst study participants, baseline hsCRP (fibrinogen) measurements were available for 1,508 (1,280) participants with MCE as the first CVD event, 5,418 (2,280) participants with IS as the first CVD event, 4,476 (2,011) participants with ICH as the first CVD event, and 5,285 (3,028) participants with no CVD event before 1^st^ January 2017 (Fig. S1**)**. Repeat measurements of hsCRP and fibrinogen were obtained in a randomly selected subset of individuals using plasma samples collected during the second resurvey in 2013–2014 (hsCRP, n = 1,296; fibrinogen, n = 639).

### Statistical analysis

For all analyses, data for hsCRP, fibrinogen, and selected biomarkers (TG, AST, ALT, GGT, Lp(a), creatinine, cystatin-C) were log-transformed. Adjusted means and proportions of baseline characteristics for cases and controls, and cross-sectional mean levels of hsCRP and fibrinogen for categories (or quintiles) of selected CVD risk factors, were standardized (as appropriate) to age, sex, study area, and case status using R package lsmeans^44^ with *P* values for association derived from linear regression models (Wald test). To assess cross-sectional associations of quantitative traits (standardized using the R *scale* function) per standard deviation (SD) of log-transformed hsCRP and fibrinogen, we used linear regression, adjusted for age and sex, and stratified by study area, combining the area-specific estimates with inverse variance-weighted fixed-effect meta-analysis using function *rma* from R package metafor^45^.

To estimate odds ratios (ORs) for the associations of hsCRP and fibrinogen with risk of incident MCE, IS and ICH, logistic regression models were used with adjustment for age, age^2^, and sex (base model), and stratification by study area with inverse variance-weighted fixed effect meta-analysis. The base model was further adjusted (individually or in combination) for body-mass index (BMI), systolic blood pressure (SBP), LDL-C, TG, and other CVD risk factors: household income (in yuan: <2500, 2500–4999, 5000–9999, 10,000–19,999, 20,000–34,999, >35,000); occupation (agriculture, factory worker, administrator/manager, professional/technical, sales and service, retired, house-wife/husband, self-employed, unemployed, other or not stated); education (no formal school, primary school, middle school, high school, technical school/college, university); standing height (meters); self-reported or screen-detected diabetes status^46^; physical activity (metabolic equivalent of task hours per day, MET h/day)^47^; smoking (never smoker, occasional smoker, ex-regular smoker, smoker); and alcohol consumption (never regular drinker, ex-regular drinker, occasional or seasonal drinker, monthly drinker, reduced intake, weekly drinker)^48^. Where models failed to converge due to very low numbers of cases, those study area(s) or strata were excluded from the analysis. The Wald χ^2^ statistic (with one degree of freedom) was calculated using function *anova.rma* from R package metafor to assess the improvement in model fit following inclusion of additional covariates. Heterogeneity and trend tests were conducted by χ^2^ tests applied to the log ORs and their SEs. For quintiles of hsCRP and fibrinogen, floating CIs were estimated using the Plummer method^49^ to provide 95% CI for all quintiles including the reference quintile, enabling inter-quintile comparisons.

For tests of the potential discriminatory utility of hsCRP for MCE, IS, and ICH, C-statistic using the area under the receiver-operating characteristic curve (AUC) was calculated and DeLong’s test was applied, using the *roc* and *roc.test* functions, respectively, from R package pROC. Likelihood ratio tests were performed using the R *anova* function. Net Reclassification Improvements were estimated using the *reclassification* function from R package PredictABEL.

Regression calibration was used to adjust for within-person variation in hsCRP and fibrinogen. Regression coefficients and predicted usual values were estimated from linear regression of log-transformed hsCRP or fibrinogen levels from resurvey samples (taken median 5 years after baseline) on the corresponding baseline values for the same participants. Quintile plots used predicted usual values for CRP and fibrinogen levels; adjusted log ORs per SD of usual log CRP and fibrinogen levels and corresponding standard errors were calculated by dividing those derived using baseline CRP and fibrinogen levels by the corresponding regression coefficient^50^.

Where stated, significance thresholds for *P* values from multiple hypothesis testing were corrected using the Bonferroni method. Analyses were performed using R version 3.5.0.

### Ethical approval and informed consent

1. **Approval*:*** Ethical approval for CKB was obtained from the University of Oxford, the Chinese Centre for Disease Control and Prevention (CCDC) and the regional CDC from the 10 study areas
2. **Accordance**: Study conducted according to the Declaration of Helsinki.
3. **Informed consent*:*** All study participants provided written informed consent for participation in CKB

## Supplementary information

Supplement.

## Data Availability

CKB data, including data used for this study, are available on application according to the CKB data access policy. Data access is overseen by an independent data access committee. Further details can be found here: https://www.ckbiobank.org/site/Data+Access
