# Publisher Correction: Systemic inflammation is associated with incident stroke and heart disease in East Asians

**DOI:** 10.1038/s41598-020-64764-0

**Published:** 2020-05-12

**Authors:** Mohd A. Karim, Christiana Kartsonaki, Derrick A. Bennett, Iona Y. Millwood, Michael R. Hill, Daniel Avery, Zheng Bian, Huaidong Du, Yu Guo, Yijian Qian, Chan Qu, Iain Turnbull, Dan Schmidt-Valle, Chunmei Wang, Canqing Yu, Jun Lv, Junshi Chen, Robert Clarke, Liming Li, Zhengming Chen, Michael V. Holmes, Robin G. Walters, China Kadoorie Biobank Collaborative Group, Rory Collins, Depei Liu, Richard Peto, Ruth Boxall, Yumei Chang, Yiping Chen, Simon Gilbert, Alex Hacker, Andri Iona, Rene Kerosi, Ling Kong, Om Kurmi, Garry Lancaster, Sarah Lewington, Kuang Lin, John McDonnell, Qunhua Nie, Paul Ryder, Sam Sansome, Paul Sherliker, Rajani Sohoni, Becky Stevens, Jenny Wang, Lin Wang, Neil Wright, Ling Yang, Xiaoming Yang, Pang Yao, Xiao Han, Can Hou, Pei Pei, Chao Liu, Zengchang Pang, Ruqin Gao, Shanpeng Li, Shaojie Wang, Yongmei Liu, Ranran Du, Liang Cheng, Xiaocao Tian, Hua Zhang, Yaoming Zhai, Feng Ning, Xiaohui Sun, Feifei Li, Silu Lv, Junzheng Wang, Wei Hou, Mingyuan Zou, Shichun Yan, Xue Zhou, Bo Yu, Yanjie Li, Qinai Xu, Quan Kang, Ziyan Guo, Dan Wang, Ximin Hu, Jinyan Chen, Yan Fu, Xiaohuan Wang, Min Weng, Zhendong Guo, Shukuan Wu, Yilei Li, Huimei Li, Ming Wu, Yonglin Zhou, Jinyi Zhou, Ran Tao, Jie Yang, Jian Su, Fang liu, Jun Zhang, Yihe Hu, Yan Lu, Liangcai Ma, Aiyu Tang, Yujie Hua, Jianrong Jin, Jingchao Liu, Zhenzhu Tang, Naying Chen, Ying Huang, Mingqiang Li, Jinhuai Meng, Rong Pan, Qilian Jiang, Jian Lan, Yun Liu, Liuping Wei, Liyuan Zhou, Ningyu Chen, Ping Wang, Fanwen Meng, Yulu Qin Sisi Wang, Xianping Wu, Ningmei Zhang, Xiaofang Chen, Weiwei Zhou, Guojin Luo, Jianguo Li, Xiaofang Chen, Xunfu Zhong, Jiaqiu Liu, Qiang Sun, Pengfei Ge, Xiaolan Ren, Caixia Dong, Hui Zhang, Enke Mao, Xiaoping Wang, Tao Wang, Xi Zhang, Ding Zhang Zhou, Gang Zhou, Shixian Feng, Ling Chang, Lei Fan, Yulian Gao, Tianyou He, Huarong Sun, Pan He, Chen Hu, Xukui Zhang, Huifang Wu, Min Yu, Ruying Hu, Hao Wang, Weiwei Gong, Meng Wang, Kaixu Xie, Lingli Chen, Dongxia Pan, Qijun Gu, Yuelong Huang, Biyun Chen, Li Yin, Huilin Liu, Zhongxi Fu, Qiaohua Xu, Xin Xu, Hao Zhang, Huajun Long, Libo Zhang

**Affiliations:** 1 Clinical Trial Service Unit & Epidemiological Studies Unit (CTSU), Nuffield Department of Population Health, University of Oxford, Oxford, United Kingdom; 2 Medical Research Council Population Health Research Unit (MRC PHRU) at the University of Oxford, Nuffield Department of Population Health, University of Oxford, Oxford, United Kingdom; 3 Chinese Academy of Medical Sciences, Beijing, China; 4 Tongxiang Centre for Disease Control and Prevention, Tongxiang, Zhejiang Province China; 5 NCDs Prevention and Control Department, Liuyang Centre for Disease Control and Prevention, Liuyang, Hunan Province China; 6 Department of Epidemiology, School of Public Health, Peking University Health Science Center, Beijing, China; 7 China National Center for Food Safety Risk Assessment, Beijing, China; 8 National Institute for Health Research Oxford Biomedical Research Centre, Oxford University Hospital, Oxford, United Kingdom; 9 Centre for Disease Control and Prevention, Qingdao Province Shandong, China; 10 Licang Centre for Disease Control and Prevention, Qingdao Province Shandong, China; 11 Centre for Disease Control and Prevention, Heilongjiang Province, Harbin, China; 12 Nangang Centre for Disease Control and Prevention, Heilongjiang Province, Harbin, China; 13 Centre for Disease Control and Prevention, Hainan Province, Haikou, China; 14 Meilan Centre for Disease Control and Prevention, Hainan Province, Haikou, China; 15 Centre for Disease Control and Prevention, Jiangsu Province, Nanjing, China; 16 Suzhou Centre for Disease Control and Prevention, Jiangsu Province, Suzhou, China; 17 Wuzhong Centre for Disease Control and Prevention, Jiangsu Province, Suzhou, China; 18 Centre for Disease Control and Prevention, Guangxi Province, Nanning, China; 19 Liuzhou Centre for Disease Control and Prevention, Guangxi Province, Liuzhou, China; 20 Centre for Disease Control and Prevention, Sichuan Province, Chengdu, China; 21 Pengzhou Centre for Disease Control and Prevention, Sichuan Province Pengzhou, China; 22 Centre for Disease Control and Prevention, Gansu Province, Lanzhou, China; 23 Maiji Centre for Disease Control and Prevention, Gansu Province Tianshui, China; 24 Centre for Disease Control and Prevention, Henan Province Zhengzhou, China; 25 Huixian Centre for Disease Control and Prevention, Henan Province Huixian, China; 26 Centre for Disease Control and Prevention, Zhejiang Province Hangzhou, China; 27 Tongxiang Centre for Disease Control and Prevention, Zhejiang Province Tongxiang, China; 28 Centre for Disease Control and Prevention, Hunan Province Changsha, China; 29 Liuyang Centre for Disease Control and Prevention, Hunan Province Liuyang, China

Correction to: *Scientific Reports* 10.1038/s41598-020-62391-3, published online 27 March 2020

In the original version of this Article, Michael V. Holmes and Robin G. Walters were omitted as jointly supervising this work. This has now been corrected in the PDF and HTML versions of the paper.

