# Supplementary material for: Systemic inflammation is associated with incident stroke and heart disease in East Asians
Source: Sci Rep. 2020 Mar 27;10:5605. doi: 10.1038/s41598-020-62391-3 (PMC7101367; doi:10.1038/s41598-020-62391-3)
Supplement: Supplementary file 1 — Supplement. [file 41598_2020_62391_MOESM1_ESM.pdf]

SUPPLEMENTAL MATERIAL

Systemic inflammation is associated with incident stroke and heart disease in East Asians

Mohd A. Karim, MBBS, MSc[1], Christiana Kartsonaki, DPhil[1,2], Derrick A. Bennett, PhD[1], Iona Y. Millwood, DPhil[1,2], Michael R. Hill, DPhil[1,2], Daniel Avery, MSc[1], Zheng Bian, MD, MSc[3] , Huaidong Du, MD, PhD[1,2], Yu Guo, MSc[3], Yijian Qian, BSc[4], Chan Qu[5], Iain Turnbull, MRCP[1], Dan Schmidt-Valle, MSc[1], Chunmei Wang, BSc[4], Canqing Yu, PhD[3], Jun Lv, PhD[6], Junshi Chen, MD[7], Robert Clarke, MD[1], Liming Li, MBBS, PhD[6], Zhengming Chen, MD, DPhil[1], Michael V. Holmes, MD, PhD*[1,2,8], Robin G. Walters, PhD*[1,2], the China Kadoorie Biobank Collaborative Group.

* shared senior authors

1. **Clinical Trial Service Unit & Epidemiological Studies Unit (CTSU)**, Nuffield Department of Population Health, University of Oxford, United Kingdom;
2. **Medical Research Council Population Health Research Unit (MRC PHRU)** at the University of Oxford, Nuffield Department of Population Health, University of Oxford, United Kingdom;
3. **Chinese Academy of Medical Sciences**, Beijing, China;
4. **Tongxiang Centre for Disease Control and Prevention,** Zhejiang Province, China;
5. **NCDs Prevention and Control Department, Liuyang Centre for Disease Control and Prevention**, Hunan Province, China;
6. **Department of Epidemiology, School of Public Health, Peking University Health Science Center**, Beijing, China;
7. **China National Center for Food Safety Risk Assessment**, Beijing, China;
8. **National Institute for Health Research Oxford Biomedical Research Centre**, Oxford University Hospital, Oxford, United Kingdom.

Corresponding Authors:
Dr Michael V. Holmes, Associate Professor,
MRC PHRU, Nuffield Department of Population Health, Big Data Institute Building, University of Oxford, Old Road Campus, Oxford, OX3 7LF, United Kingdom.
E-mail: michael.holmes@ndph.ox.ac.uk
Tel: +44 (0)1865 743644

AND

Dr Robin G. Walters, Senior Scientist,
CTSU, Nuffield Department of Population Health, Big Data Institute Building, University of Oxford, Old Road Campus, Oxford, OX3 7LF, United Kingdom.
E-mail: robin.walters@ndph.ox.ac.uk
Tel: +44 (0)1865 743977

Table S1: Definitions of Case/Control and exclusions used with corresponding ICD:10 codes (where appropriate)

Category	Definition/Disease (ICD-10 code)/Procedure
Study population (nested case-control study)	• No self-reported history of transient ischaemic attack, ischaemic heart disease, stroke, or cancer at baseline<br>• No statin use at baseline<br>• Clinical biochemistry measurements available
Controls	• No reported CVD event (ICD-10: I00-I09, I16-I25, I27-I88, I95-I99; I10-I15) up to 1st Jan 2017.
Major Coronary Events (MCE)	• First CVD event:<br>o Ischaemic heart disease (ICD-10: I20-I25) (when fatal)<br>o Acute myocardial infarction (ICD-10: I21)<br>o PCI/CABG/stent procedure
Ischemic stroke (IS)	• First CVD event:<br>o Cerebral infarction, including sequelae (ICD-10: I63, I69.3)
Intracerebral haemorrhage (ICH)	• First CVD event:<br>o Non-traumatic intracerebral haemorrhage , including sequelae (ICD-10: I61, I69.1)
Case Exclusions	• First CVD event (ICD-10: I00-I09, I16-I19, I27-I88 [except I61, I63, I69.1, I69.3], I95-I99; I10-I15) is not a case<br>• More than one case event on the same day*

* Note: we used an individuals' first event to define their CVD status, i.e. if an individual had an MCE as their first CVD and subsequently had an IS, they contributed to the MCE analysis only (as a case). When an individual experienced more than one discrete CVD event on the same day, they were excluded from the analyses. Of the 12,886 total CVD cases, this affected only 404 cases (~3% of total).

Table S2: Geometric mean and 95% confidence interval (CI) of C-reactive protein (CRP) and Fibrinogen by key baseline characteristics

Baseline traits	N	CRP, mg/l[a]	*P* †	N	Fibrinogen, g/l [a]	*P* †
**Age Categories, years[b]**						
30-49	4024	0.76 (0.73-0.78)	1.16×10^{-126}	3279	2.70 (2.68-2.72)	5.42×10^{-307}
50-59	5387	1.01 (0.98-1.04)		2518	3.06 (3.03-3.08)	
60 and over	7275	1.26 (1.23-1.29)		2801	3.31 (3.29-3.34)	
**Gender[c]**						
Male	8463	1.06 (1.03-1.09)	0.30	4426	2.93 (2.90-2.95)	8.00×10^{-56}
Female	8224	1.04 (1.01-1.07)		4173	3.15 (3.12-3.17)	
**Region[d]**						
Urban	4987	1.18 (1.14-1.23)	5.32×10^{-18}	2486	3.01 (2.98-3.04)	5.03×10^{-02}
Rural	11700	0.99 (0.97-1.02)		6113	3.04 (3.02-3.06)	
**Education**						
Below Middle School	10192	1.06 (1.03-1.09)	0.52	4774	3.04 (3.02-3.06)	0.25
Middle school and above	6495	1.04 (1.00-1.08)		3825	3.02 (2.99-3.05)	
**Income (annual)**						
<10,000 yuan	6480	1.03 (1.00-1.07)	0.25	3227	3.03 (3.01-3.06)	0.81
≥10,000 yuan	10207	1.06 (1.03-1.09)		5372	3.03 (3.01-3.05)	
**Occupation**						
Non/Semi-manual*	6904	1.18 (1.14-1.23)	9.02×10^{-19}	3198	3.08 (3.05-3.11)	2.49×10^{-05}
Manual**	9783	0.97 (0.94-1.00)		5401	3.01 (2.98-3.03)	
**Drinking (Males)**						
Less than weekly	5898	1.02 (0.98-1.06)	0.68	3044	2.98 (2.95-3.02)	1.51 x 10-^{16}
Weekly	2565	1.04 (0.98-1.09)		1382	2.80 (2.76-2.84)	
**Smoking (Males)**						
Never smokers	1100	0.94 (0.87-1.01)	8.77×10^{-03}	560	2.97 (2.92-3.03)	5.33×10^{-02}
Ever smokers	7363	1.04 (1.00-1.08)		3866	2.92 (2.89-2.95)	
**Diabetes**						
No	15238	1.00 (0.98-1.03)	6.12×10^{-46}	7947	3.02 (3.00-3.04)	1.26×10^{-12}
Yes	1449	1.58 (1.49-1.68)		652	3.21 (3.15-3.26)	
**BMI (kg/m^2)**						
<22.0	5849	0.78 (0.75-0.80)	9.45×10^{-218}	2946	3.01 (2.99-3.04)	5.86×10^{-06}
22.0 - 24.9	5313	0.97 (0.94-1.00)		2748	3.00 (2.97-3.03)	
≥25.0	5525	1.50 (1.45-1.55)		2905	3.07 (3.05-3.10)	
**SBP (mmHg)** [e]						
<120.0	3125	0.85 (0.81-0.89)	9.34×10^{-34}	1845	2.99 (2.95-3.02)	2.09×10^{-07}
120.0 - 139.9	5496	1.01 (0.98-1.05)		2922	3.02 (2.99-3.05)	
≥140.0	8066	1.15 (1.12-1.19)		3832	3.06 (3.04-3.09)	
**LDL-cholesterol[†] (mg/dL)[f]**						
< 80.0	5942	0.92 (0.89-0.96)	3.12×10^{-40}	3281	2.92 (2.90-2.95)	1.60×10^{-51}
80 – 99.9	5020	1.01 (0.97-1.05)		2632	3.02 (3.00-3.05)	
≥100	5725	1.22 (1.18-1.26)		2686	3.16 (3.13-3.19)	
**TG (mg/dL)[f]**						
<110.0	5640	0.90 (0.87-0.93)	6.05×10^{-47}	2882	3.02 (2.99-3.05)	9.02×10^{-02}
110 – 179.9	5360	1.02 (0.98-1.05)		2753	3.05 (3.02-3.08)	
≥180.0	5687	1.24 (1.20-1.28)		2964	3.03 (3.00-3.05)	
**Physical activity (MET hours/day)**						
<10.0	5218	1.22 (1.17-1.26)	1.42×10^{-27}	2363	3.11 (3.08-3.14)	1.45×10^{-07}
10.0 – 14.9	3366	1.12 (1.07-1.17)		1632	3.05 (3.01-3.08)	
≥15.0	8103	0.95 (0.92-0.98)		4604	3 (2.97-3.02)	
**Standing height of males (m)**						
<1.60	2255	1.04 (0.98-1.10)	0.93	1026	2.92 (2.87-2.97)	0.85
1.60 - 1.64	2508	1.02 (0.97-1.07)		1260	2.93 (2.89-2.97)	
1.65 – 1.69	2107	1.02 (0.97-1.08)		1137	2.91 (2.87-2.95)	
>1.70	1593	1.04 (0.97-1.11)		1003	2.93 (2.89-2.98)	
**Standing height of females (m)**						
<1.50	2495	1.06 (1.01-1.12)	4.41×10^{-02}	1154	3.15 (3.10-3.19)	2.13×10^{-02}
1.50 - 1.54	2656	1.08 (1.03-1.14)		1321	3.17 (3.13-3.21)	
1.55 – 1.59	2044	1.09 (1.03-1.15)		1098	3.13 (3.09-3.18)	
≥1.60	1029	0.96 (0.89-1.03)		600	3.09 (3.03-3.14)	

BMI: Body mass index; SBP: Systolic Blood Pressure; LDL: Low-density lipoprotein; TG: Triglycerides

[a]Values are geometric mean (95% CI) standardised for age, sex, study area and case status unless otherwise stated

[b]Standardised for sex, study area and case status only

[c]Standardised for age, study area and case status only

[d]Standardised for age, sex and case status only

[e]Categorised based on global standard criterion for hypertension

[f]To convert LDL-C (TG) from mg/dL to mmol/L, divide by 38.67 (88.57).

†P value here represents the association of the traits (in their continuous form, where possible) with CRP, adjusted for age, sex, study area and case status unless otherwise stated.

*Includes white collar jobs, retired, housewife/husband, self-employed, un-employed or others/not stated

**Includes farmers and factory workers

[†]Low-density lipoprotein (LDL) cholesterol was directly measured (and not estimated using the Friedewald equation).

Table S3: Discriminatory performance

Vascular endpoint	Area under the Receiver Operating Characteristic curve (95%CI)			LRT** P-value
	**Model 1** (age, sex, region, smoking, diabetes, BMI, SBP, LDL, HDL, TG)	**Model 2** (Model 1 + CRP)	P-value*	
**Major Coronary Events**	0.7181 (0.7028, 0.7334)	0.7251 (0.7101, 0.7402)	1.82×10^{-3}	1.21×10^{-13}
**Ischemic stroke**	0.7816 (0.773, 0.7902)	0.7822 (0.7736, 0.7908)	0.095	1.75×10^{-4}
**Intracerebral hemorrhage**	0.7379 (0.728-0.7477)	0.7391 (0.7293-0.7489)	0.075	9.54×10^{-7}

*P-value from DeLong's test for two correlated ROC curves

**Likelihood ratio test

Table S4: Net Reclassification Improvement for A) MCE, B) IS, and c) ICH

A) MCE

	Reclassified predicted risk (with CRP)				N reclassified as		
**Predicted risk (without CRP)**	< 5%	5 to < 10%	10 to < 20%	≥ 20%	increased risk	reduced risk	Net correctly reclassified: N (Prop.)
MCE (n = 1508)							
< 5%	5	2	0	0	85	94	-9 (-0.0060[a])
5 to < 10%	4	63	19	0			
10 to < 20%	0	31	302	64			
≥ 20%	0	0	59	959			
Controls (n = 5285)							
< 5%	75	10	1	0	385	600	215 (0.041[b])
5 to < 10%	62	621	114	3			
10 to < 20%	0	267	1910	257			
≥ 20%	0	0	271	1694			
Total correctly reclassified							206 (0.035[c])
**Net reclassification improvement (95% CI)**							0.035 (0.014 - 0.056]) p-value = 0.0011

[a]NRI_{event} = P(up|event) - P(down|event)

[b]$NRI_{non\text{-}event}$ = P(down|nonevent) -P(up|nonevent)

[c]NRI = NRI_{event} + $NRI_{non\text{-}event}$

B) IS

	Reclassified predicted risk				N reclassified as		
**Predicted risk (without CRP)**	< 5%	5 to < 10%	10 to < 20%	≥ 20%	increased risk	reduced risk	Net correctly reclassified (Prop.)
IS (n = 5418)							
< 5%	0	0	0	0	25	22	3 (0.00055[a])
5 to < 10%	0	11	2	0			
10 to < 20%	0	1	141	23			
≥ 20%	0	0	21	5219			
Controls (n = 5285)							
< 5%	0	0	0	0	55	109	54 (0.010[b])
5 to < 10%	1	102	13	0			
10 to < 20%	0	14	755	42			
≥ 20%	0	0	94	4264			
Total correctly reclassified							57 (0.011[c])
**Net reclassification improvement (95% CI)**							0.011 (0.0054 - 0.016) p-value = 8.0×10^{-5}

[a]NRI_{event} = P(up|event) - P(down|event)

[b]$NRI_{non\text{-}event}$ = P(down|nonevent) -P(up|nonevent)

[c]NRI = NRI_{event} + $NRI_{non\text{-}event}$

C) ICH

	Reclassified predicted risk				N reclassified as		
**Predicted risk (without CRP)**	< 5%	5 to < 10%	10 to < 20%	≥ 20%	increased risk	reduced risk	Net correctly reclassified (Prop.)
ICH (n = 4476)							
< 5%	0	0	0	0	18	25	-7 (-0.0016[a])
5 to < 10%	0	1	1	0			
10 to < 20%	0	0	103	17			
≥ 20%	0	0	25	4329			
Controls (n = 5285)							
< 5%	0	0	0	0	52	94	42 (0.0080[b])
5 to < 10%	0	25	2	0			
10 to < 20%	0	4	590	50			
≥ 20%	0	0	90	4524			
Total correctly reclassified (%)							35 (0.0064[c])
**Net reclassification improvement (95% CI)**							0.0064 (0.0011 - 0.012) p-value = 0.019

[a]NRI_{event} = P(up|event) - P(down|event)

[b]$NRI_{non-event}$ = P(down|nonevent) -P(up|nonevent)

[c]NRI = NRI_{event} + $NRI_{non-event}$

Figure S1: Flowchart of participant selection

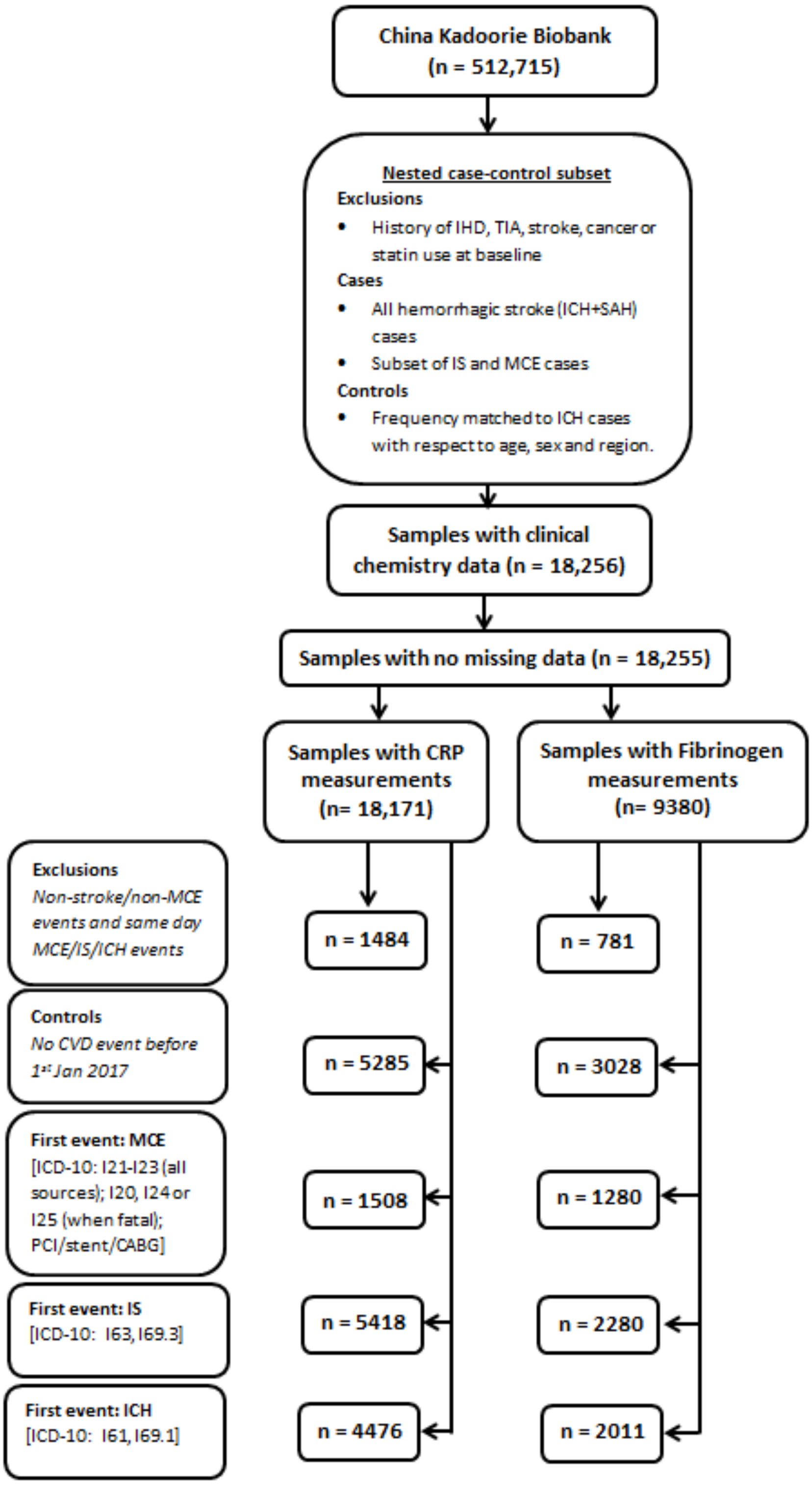

CRP: C-reactive protein; CVD: Cardiovascular disease; ICD: International Classification of Diseases; ICH: Intracerebral haemorrhage; IHD: Ischaemic heart disease; IS: Ischaemic stroke; MCE: Major Coronary Event; TIA: transient ischaemic attack;

Figure S2: Cross-sectional associations of CRP and Fibrinogen in controls with selected biomarkers

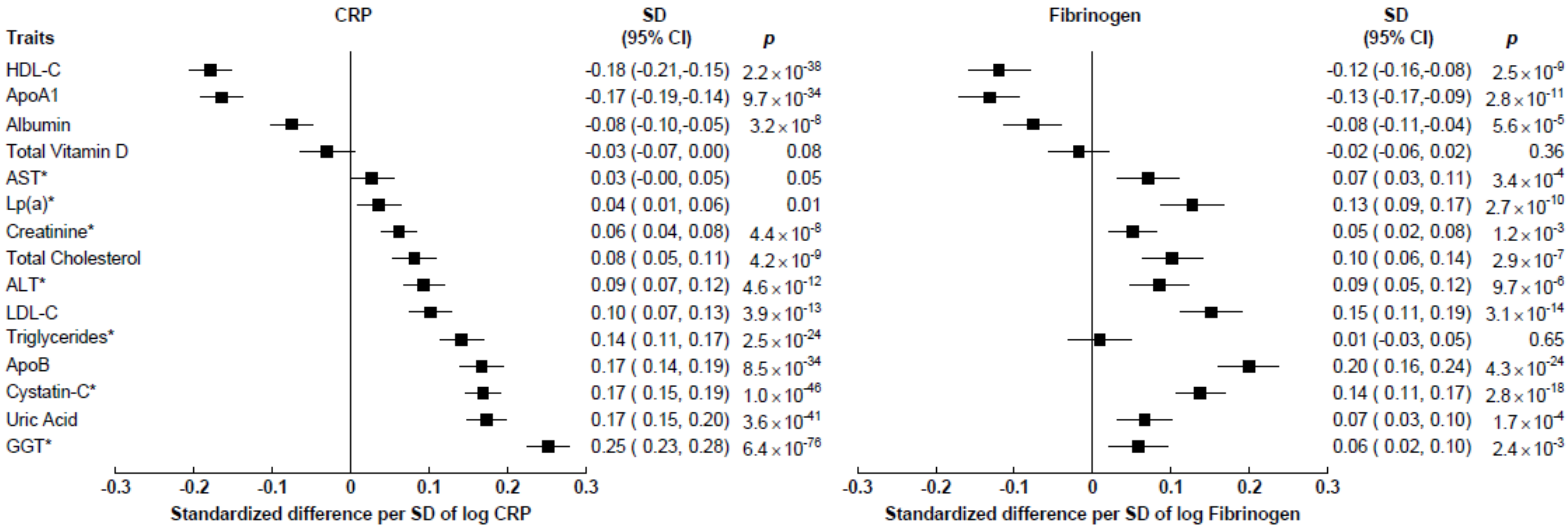

CRP: C-reactive protein; HDL-C: High-density lipoprotein cholesterol; ApoA1: Apolipoprotein A1; AST: Aspartate transaminase; Lp(a): Lipoprotein(a) ; ALT: Alanine transaminase; LDL-C: Low-density lipoprotein cholesterol; ApoB: Apolipoprotein B; GGT: Gamma-glutamyl transferase

Note: All biomarkers standardized prior to analysis
*Biomarkers which were also log-transformed before standardization

Figure S3: Risk of (A) Major Coronary Events, (B) Ischemic Stroke and (C) Intracerebral Hemorrhage per standard deviation (SD) of usual log-transformed Fibrinogen levels

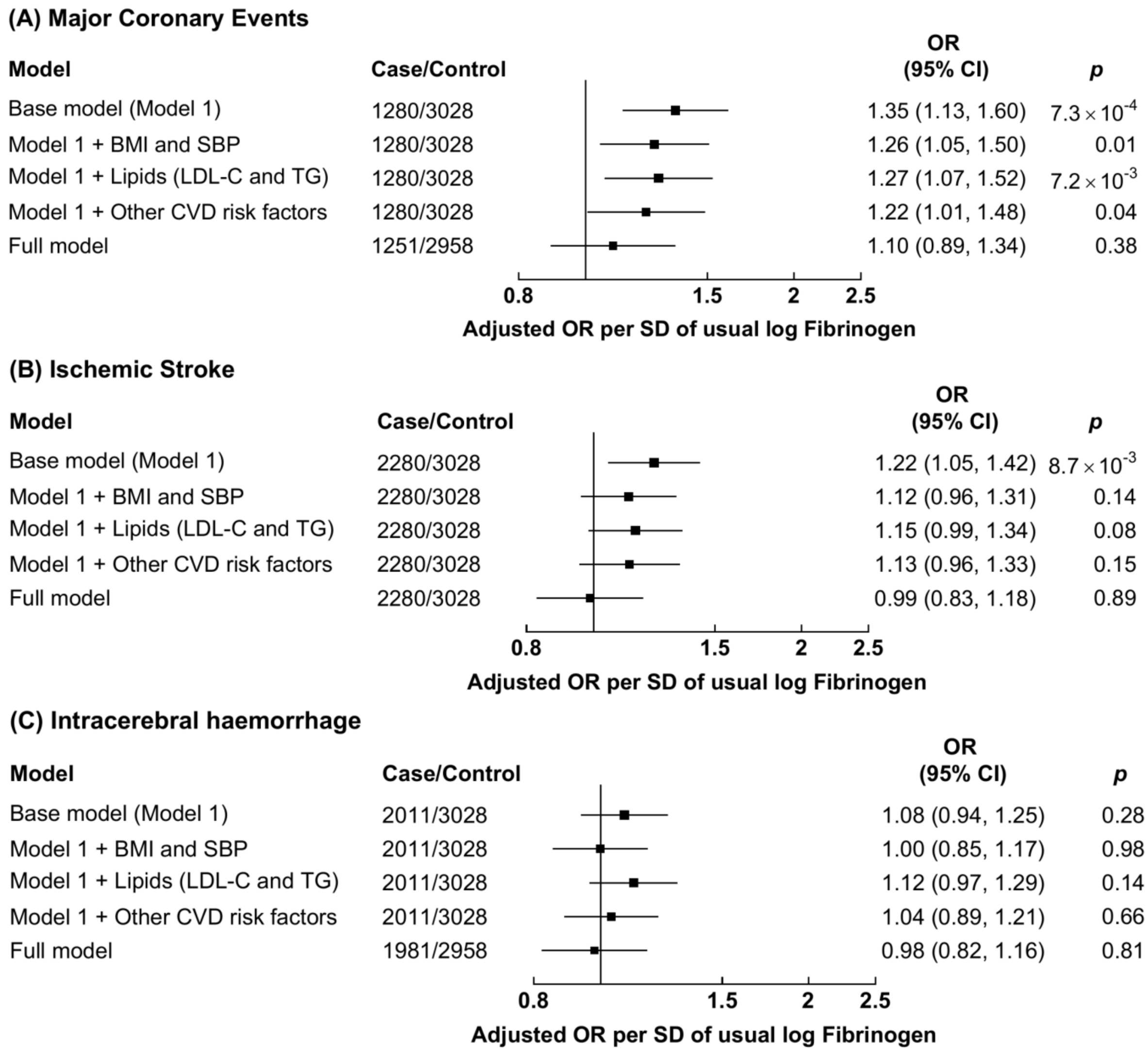

Base model includes age, age^2 and sex.
Other CVD Risk Factors: income, occupation, education, diabetes, physical activity, standing height, smoking, and alcohol
Full model includes base model, body-mass index (BMI), systolic blood pressure (SBP), low-density lipoprotein cholesterol (LDL-C), triglycerides (TG) and other CVD risk factors;

Note: For each Model, models were fitted separately for each of the 10 study areas and estimates were meta-analyzed; study areas were excluded when models failed to converge (2 models out of 150 models)

Figure S4: Progressive adjustment of (A) CRP and (B) Fibrinogen for Major Coronary Events, Ischemic Stroke and Intracerebral hemorrhage

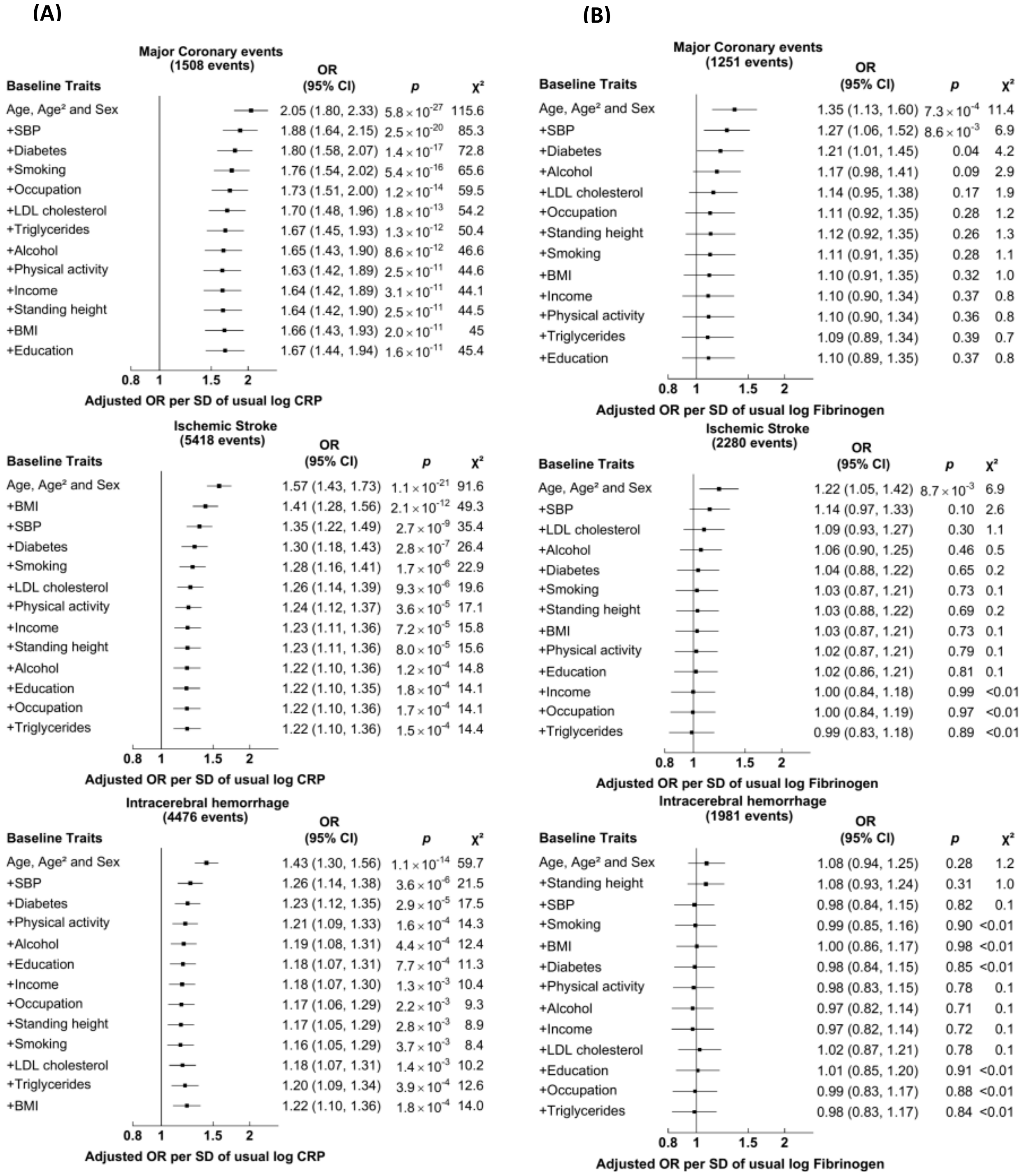

Note: Models were fitted separately for each of the 10 study areas for each covariate and estimates were meta-analyzed; for each covariate, study areas were excluded when models failed to converge (2 models out of 780 models). Progressive adjustment used at each step the covariate giving the largest change in x^2.

Figure S5: Risk of Major Coronary Events, Ischemic Stroke and Intracerebral Hemorrhage per standard deviation (SD) of usual Fibrinogen levels by subgroups

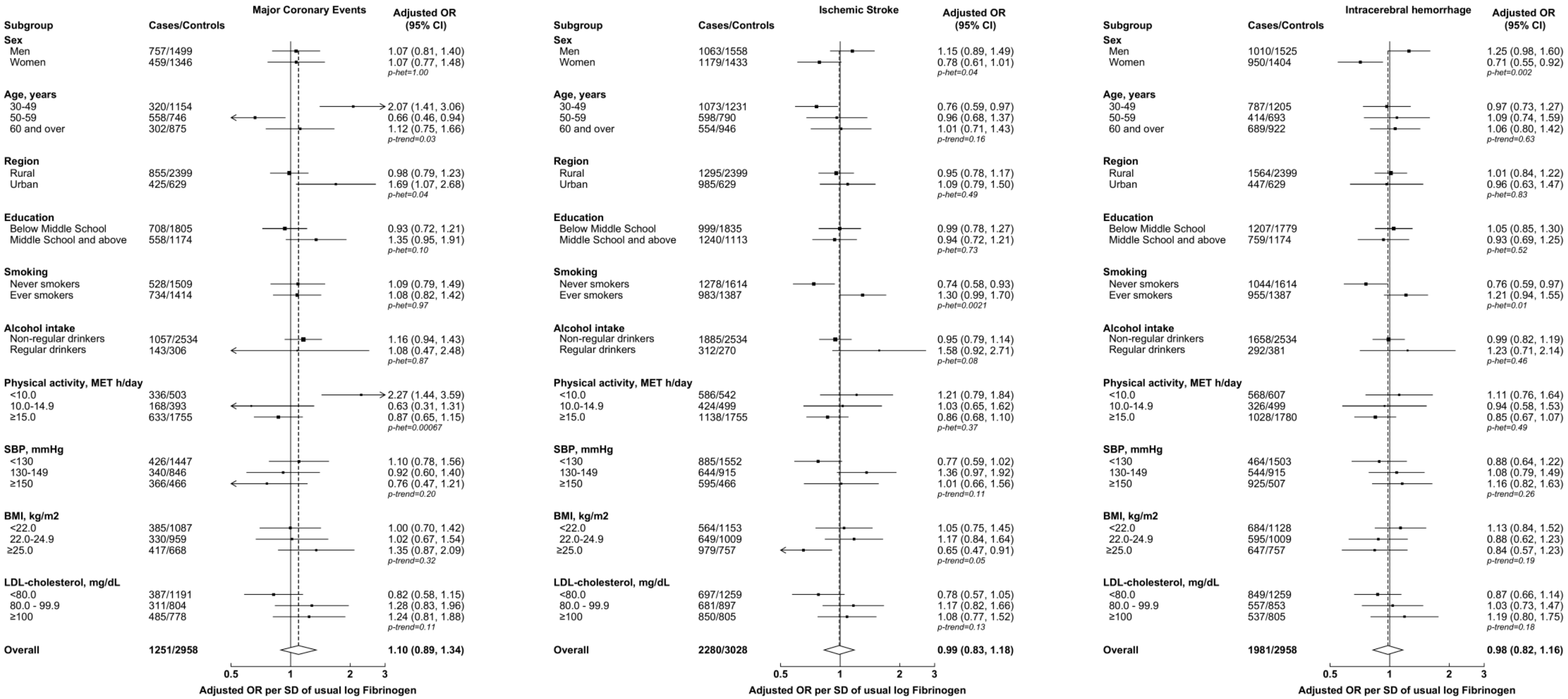

Note: Models were adjusted (where appropriate) for age, age^2 and sex (base model) and additionally for income, occupation, education, SBP, BMI, diabetes, physical activity, standing height, smoking, alcohol, LDL-C and TG. The models were fitted separately for each of the 10 study areas in each subgroup strata and estimates were meta-analyzed; in each subgroup strata, study areas were excluded when models failed to converge (141 models out of 750 models). Error bars represent 95% confidence intervals (CI). The sizes of the boxes are proportional to the inverse of the variance of the log ORs. The dotted line represents the overall adjusted OR. The open diamond represents the overall adjusted OR and its 95% CI. MET, metabolic equivalent of task; *p-het*, p-value for heterogeneity; *p-trend*, p-value for trend.
